# Socio-economic inequalities in high blood pressure and additional risk factors for cardiovascular disease among older individuals in Colombia: Results from a nationally representative study

**DOI:** 10.1371/journal.pone.0234326

**Published:** 2020-06-09

**Authors:** Philipp Hessel, Paul Rodríguez-Lesmes, David Torres

**Affiliations:** 1 University of the Andes, Alberto Lleras Camargo School of Government, Bogotá, Colombia; 2 University Del Rosario, Faculty of Economics, Bogotá, Colombia; Universidade Federal de Pelotas, BRAZIL

## Abstract

**Background:**

Studies in high-income countries have documented a consistent gradient between socio-economic status (SES) and high blood pressure (HBP), a key risk factor for cardiovascular disease (CVD). However, evidence from Latin American countries (LA) remains comparatively scarce and inconclusive.

**Data:**

Data for 3,984 individuals came from a nationally representative survey of individuals aged 60 years or above in Colombia (*Encuesta de Salud*, *Bienestar y Envejecimiento*) (SABE) conducted in 2015. SES was measured by educational achievement and household assets. CVD risk factors included objectively measured HBP and body mass index (BMI), as well as behaviors (smoking, alcohol consumption, fruit and vegetables intake, and physical activity).

**Methods:**

Bivariate methods and multivariate regression models were used to assess associations between SES with HBP as well as additional risk factors for CVD.

**Results:**

Individuals with lower SES have significantly higher risk of suffering from HBP. Compared to those with no formal education, individuals with secondary or post-secondary education have a 37% lower risk of HBP (odds ratio [OR] = 0.63, P-value<0.001). Being in the highest asset quartile (most affluent) is associated with a 44% lower risk (OR = 56, P-value = 0.001) of HBP compared to those in the lowest asset quartile (most deprived). Individuals with lower SES are more likely to smoke, not engage in regular physical activity and not regularly consume fruits or vegetables. In contrast, individuals with higher SES are more likely to consume alcohol and, those with more assets, more likely to be obese.

**Conclusions:**

Among older Colombians there exists a marked SES gradient in HBP as well as several additional risk factors for CVD. The results highlight the importance of a public health approach towards HBP and additional CVD risk factors that takes into account the specific conditions of older individuals, especially among disadvantaged groups.

## Introduction

The prevalence of chronic non-communicable diseases (NCD) is increasing around the world, representing a substantial share of health care expenditures as well as morbidity and mortality in low and middle-income countries (LMICs). [1] Besides globalization and urbanization, population aging has been singled out as a key driving factors of increasing prevalence of NCDs. [2,3] This particularly applies to Latin America (LA), a region that is not only projected to soon be the world’s most urbanized but also one of the most rapidly aging ones. NCDs have surpassed infectious diseases as the primary cause of death in the region, [4,5] with cardiovascular disease (CVD) being the leading cause of death in the. [6,7] High blood pressure (HBP), is the leading risk factor behind the increase in CVD, [8] with an estimated average population prevalence of 20%. [9]

A critical feature of the rising epidemic of NCDs is the presence of growing socio-economic inequalities by which the disease burden is becoming increasingly concentrated in disadvantaged groups. [10,11] The existence and extent of socio-economic status (SES) inequalities in NCDs, in general, and CVD more specifically, has been extensively studied in high-income countries—overwhelmingly showing consistent and substantial SES gradients in CVD and key risk factors, including HBP. [12–15]

In contrast, evidence on SES inequalities in HBP and additional risk factors for CVD in LA remains comparatively scarce and conflicting. Existing studies among older individuals in LA suggest that SES is either associated with HBP, [16] only inconsistently associated with HBP, [17,18] or even not associated with the latter at all. [19] This finding is generally puzzling, [18] given that LA is one of the most unequal regions of the world, with levels of inequality much higher than in Europe or the United States. A potential explanation for the absence of a consistent SES gradient in CVD and its key risk factors in LA is that countries are at different stages of an epidemiological transition in which the main burden of CVD (risk factors) shifts from being concentrated among individuals with higher SES to being concentrated mainly among those with lower SES. [20] Another explanation for the inconsistent findings regarding the SES gradient in CVD and CVD risk factors in LA may be due to the small number of related studies, specifically those based on representative data and valid measures of SES and CVD (risk factors). [20,21]

Using representative data for individuals aged 60 years and above from the *Encuesta de Salud*, *Bienestar y Envejecimiento* (Study of Health, Well-being, and Aging, or SABE according to its Spanish acronym) this study systematically assesses the relation between SES (measured by educational achievement as well as household assets), objectively measured HBP and additional risk factors for CVD including obesity, physical activity, fruits and vegetable intake, smoking and alcohol consumption.

## Materials & methods

### Data

Data from this study came from the SABE 2015 survey. [22] The latter is a nationally representative survey for the non-institutionalized population aged 60 years and above in Colombia. The total sample size is 23,694 individuals from 244 municipalities and all departments of the country. A proxy respondent aided 4,690 of the 23,694 individuals in the sample. Individuals were included in the survey if they were aged 60 years or more, provided written consent and were able to communicate with the interview team clearly. The data contain detailed information on respondent’s health, anthropometric measures, demographic characteristics, and SES. [22–25] Individuals were selected for face-to-face computer-assisted personal interviews (CAPI) using a multi-stage cluster sampling procedure, using municipalities as the primary sampling unit. The overall response rate was 70%. [23] Rigorous quality-control and—assurance procedures were applied that included verifying information collected in the interviews by telephone using available register data provided by the National Statistical Administrative Department (DANE). Ethics committees of both University of Caldas and University of Valle reviewed and approved the study protocol. [23]

The SABE data can be requested from the Colombian Ministry of Health. Although the user agreement does not permit sharing the data directly, under the following link we include the data request form, detailed instructions on how the data can be requested, as well as complete replication materials (https://github.com/PhilippHessel/Colombia_SES_CVD_OlderIndividuals).

The present study is based on a subsample of individuals that were randomly selected to have their blood pressure measured. A total of 4,324 individuals that were not aided by a proxy respondent had their blood pressure successfully measured. Of the latter, 3,999 successfully had their height and weight assessed. The main analyses of this study are based on 3,984 individuals that successfully had their blood pressure as well as height and weight, and that had no missing on any of the included variables (see Table 1).

Further below, we discuss potential bias of the results due to non-random failure to successfully participate in the assessment of blood pressure.

**Table 1:** Descriptive statistics.

Risk factors for cardiovascular disease	Men		Women	
	*N (Mean)*	*% (SD)*	*N (Mean)*	*% (SD)*
**Blood pressure**				
Systolic blood pressure (SBP) high (> = 140 mm Hg) (yes)	606	35.58	656	28.76
Diastolic blood pressure (DBP) high (> = 90 mm Hg) (yes)	158	9.28	88	3.86
High blood pressure (HBP) = SBP or DBP high (yes)	622	36.52	669	29.33
**Additional risk factors**				
Obese (yes)	232	13.62	659	28.89
Smoker (yes)	255	14.97	171	7.5
Alcohol consumption (yes)	421	24.72	132	5.79
Fruits or vegetables consumption (no)	613	36.00	662	29.02
Physical activity (no)	1,244	73.05	1,865	81.76
**Socio-economic status**				
**Education**				
None	224	13.15	288	12.63
Primary	1,010	72.46	1,394	61.11
Secondary or Post-Secondary	469	27.54	599	26.26
**Assets Index** (0–16)	(7.75)	(3.56)	(8.03)	(3.32)
**Covariates**				
Age	(69.55)	(7.00)	(68.84)	(6.85)
Region				
Central	455	26.72	709	31.08
Bogotá	357	20.96	484	21.22
Pacific	352	20.67	451	19.77
Atlantic	274	16.09	312	13.68
Eastern	249	14.62	312	13.68
Orinoquia & Amazonas	16	0.94	16	0.7
N	1,703		2,281	

Abbreviations: SD = standard deviation; mm Hg = millimeters of mercury; SBP = systolic blood pressure; DBP = diastolic blood pressure; HBP = high blood pressure. Authors’ own calculations based on data from the *Encuesta de Salud*, *Bienestar y Envejecimiento* (SABE) study. The sample includes men and women aged at least 60 years or above that participated in the measure of blood pressure had no missing values of any of the remaining covariates. HBP is defined as having either systolic blood pressure >= 140 mm Hg or having diastolic blood pressure >= 90 mm Hg.

### Measures

#### Measurement of socio-economic status

SES was measured in terms of self-reported educational achievement as well as household assets.

Based on originally 11 response categories for the highest educational level achieved, we re-classified individuals into three groups, distinguishing between the following categories: 1) none, 2) primary (incomplete or complete), 3) secondary or post-secondary (complete or incomplete).

Assets were measured based on a summative index indicating whether individuals’ households possessed a functioning version of each of the following [17] items or not: radio, television, speakers, DVD player, fan, computer, cell phone, fridge, blender, washing machine, electric or gas oven, microwave, vacuum cleaner, shower with warm water, ambient heater / air conditioning, internet access, cable television. The index had an approximately normal distribution with an average of available items of 7.9. Based on the summative index we classified each individual into quartiles with the 1st (most deprived) quartile ranging between 0 to 5 items, the 2nd quartile ranging from 5 to 8 items, the 3rd quartile ranging from 8 to 10 items, and the 4th (most affluent) quartile ranging from 10 to 16 items.

#### Measurement of high blood pressure

For assessing prevalence of HBP we relied on assessments of both systolic blood pressure (SBP) as well as diastolic blood pressure (DBP). Both were assessed using an electronic manometer (HEM-7113, Omron Healthcare Co., Ltd., Kyoto, Japan) and recorded in millimeter of mercury (mm Hg). The obtained values were recorded after five minutes of resting in the sitting position, with three consecutive measures being obtained, while waiting at least 30 seconds between each measure. Following the manual of the Colombian Ministry of Health, [26] individuals were classified as suffering from HBP if both the second and third assessment of the right arm indicated SBP of at least 140 mm Hg or higher, or both the second and third assessment of the right arm indicated DBP of at least 90 mm Hg or higher.

#### Measures of additional risk factors for cardiovascular disease

Prevalence of obesity was measured based on anthropometric measures of body height and weight using a portable stadiometer (SECA 213) and an electronic scale (Kendall platform scale graduated). Based on the measures of height and weight we calculated individuals’ body mass index (BMI) in units of kilograms (kg) by the square of body height in meters (m^2^). Individuals were classified as being overweight if their corresponding BMI was > 30 kg/m^2^. Additional risk factors for CVD included in this study include excessive alcohol consumption, low consumption of fruits and vegetables, lack of physical activity, smoking as well as being overweight. Alcohol consumption was operationalized in the form of a binary variable distinguishing between respondents not consuming any alcohol and those drinking alcohol at least once a week or more often. Consumption of fruits or vegetables was measured in the form of a binary variable distinguishing individuals that consume fruits or vegetables at least twice a day from those that consume fruits or vegetables less frequently. Lack of physical activity was measured by a binary variable capturing whether or not a person participated at least three times per week in any sports or physical activity. Smoking was operationalized by distinguishing individuals who currently smoked with those that either never smoked or those that had smoked in the past.

#### Covariates

Covariates include age (in years), gender as well as region of residence. The latter represent the five census regions of Colombia (Atlantic region, Capital region of Bogotá, Central region, Eastern region, the regions of Orinoquia and Amazonas, and the Pacific region).

### Statistical analyses

**Fig 1:**
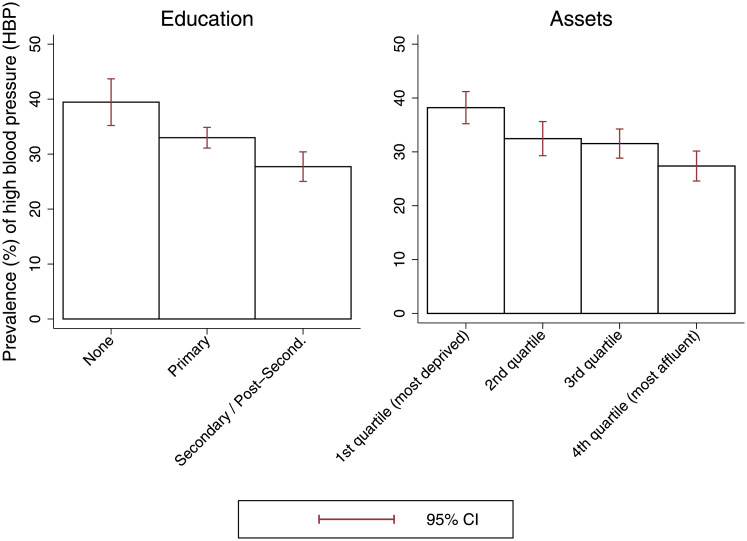
Prevalence of high blood pressure according to socio-economic status among older individuals in Colombia. Authors’ own calculations based on data from the *Encuesta de Salud*, *Bienestar y Envejecimiento* (SABE) study. The figure shows prevalence of high blood pressure (HBP) according to socio-economic status (SES), including 95% confidence intervals (CI), among individuals aged 60 years or older.

**Fig 2:**
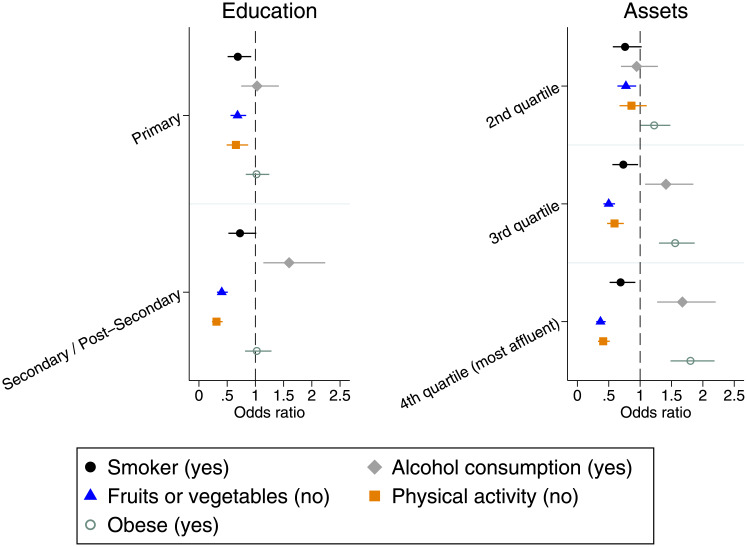
Associations between risk factors for cardiovascular disease and socio-economic status among older individuals in Colombia. Authors’ own calculations based on data from the *Encuesta de Salud*, *Bienestar y Envejecimiento* (SABE) study including individuals aged 60 years or above. The figure shows the association between socio-economic status (SES) with different risk factors for cardiovascular disease (CVD). Results are shown in terms of odds ratios (OR) and refer to the difference in relative risk of each SES category in comparison with the lowest category in terms of either education (none) or assets (1st quartile—most deprived). Results were obtained from multivariate logistic regressions that controlled for age, gender and region of residence. Detailed results are shown in S1 Appendix Supplementary Table A2.

Bivariate methods and multivariate regression models were used to assess associations between SES with HBP as well as additional risk factors for CVD. In a first step, we present each variable included in the analyses in terms of its means *±* standard deviations (SD) or the distribution of its sub-categories in percent (%) (depending on the measurement scale), separately by gender (Table 1). Although Table 1 suggests that there are differences between men and women with regard to HBP and SES, when including an interaction term between SES (measured by either educational levels or asset quartiles) and gender in the multivariate regression models (that control for age and region of residence), no significant interactions existed. In consequence, we do not present the multivariate analyses separate by gender, but control for gender as covariate. Secondly, we graphically show prevalence of HBP according to SES (Fig 1). In a third step, we use multivariate logistic regression models to assess associations between HBP and SES, controlling for age, gender and region of residence (Table 2). Finally, multivariate logistic regressions were used to assess associations between SES with additional behavioral risk factors for CVD (Fig 2 & in S1 Appendix Supplementary Table A2).

**Table 2:** Logistic regressions of high blood pressure on socio-economic status among older individuals in Colombia.

	Education			Assets		
	*OR*	*95% CI*	*P-value*	*OR*	*95% CI*	*P-value*
**Education**						
None (reference)						
Primary	0.776	(0.634, 0.950)	0.014			
Secondary or Post-Secondary	0.626	(0.498, 0.788)	<0.001			
**Assets**						
Quartile 1 (most deprived) (reference)						
Quartile 2				0.761	(0.625, 0.926)	0.006
Quartile 3				0.718	(0.597, 0.863)	<0.001
Quartile 4 (most affluent)				0.562	(0.461, 0.685)	<0.001
Female (ref. Male)	0.735	(0.642, 0.842)	<0.001	0.745	(0.650, 0.853)	<0.001
**Region**						
Central (ref.)						
Bogotá	1.560	(1.289, 1.888)	<0.001	1.662	(1.370, 2.017)	<0.001
Pacific	1.111	(0.911, 1.354)	0.298	1.063	(0.871, 1.298)	0.546
Atlantic	1.140	(0.916, 1.418)	0.241	1.156	(0.929, 1.438)	0.193
Eastern	1.013	(0.811, 1.267)	0.907	0.971	(0.776, 1.216)	0.798
Orinoquia & Amazonas	1.172	(0.553, 2.485)	0.678	1.198	(0.565, 2.538)	0.637
**Age**						
60–64 (ref.)						
65–69	1.319	(1.099, 1.585)	0.003	1.357	(1.130, 1.629)	0.001
70–74	1.588	(1.302, 1.938)	<0.001	1.633	(1.339, 1.991)	<0.001
75–100	1.939	(1.606, 2.342)	<0.001	1.993	(1.653, 2.403)	<0.001
Constant	0.639	(0.459, 0.889)	0.008	0.622	(0.462, 0.836)	0.002
Joint significance test for SES	<0.001			<0.001		

Abbreviations: OR = odds ratio; CI = confidence interval.

Authors’ own calculations based on data from the *Encuesta de Salud*, *Bienestar y Envejecimiento* (SABE) study including individuals aged 60 years or above. The table shows the results of multivariate logistic regressions of high blood pressure (HBP) on SES and covariates. HBP is defined as having either systolic blood pressure > = 140 mm Hg or diastolic blood pressure > = 90 mm Hg. The joint significance test for SES refers to a Wald test assessing the null hypothesis that all SES categories, either for educational levels or asset quartiles, are jointly equal to zero.

## Results

The sample (Table 1) is predominantly female 57%, and the average age of men 69.5 years and of women 68.8 years. Most of the surveyed individuals have completed some years of education at the primary level (72.5% of men and 61% of women). Respondents possessed on average 8 of the 17 assets that were included in the index. The largest share of respondents came from the Central region (≈29%) or the capital city Bogotá (≈21%). Thirty-six-point five percent of men and 29% of women had HBP. Thirteen percent of men and 30% of women were classified as obese. Around fifteen percent of men and 7.5% of women in the sample were current smokers. Around twenty five percent of men and 6% of women consumed any alcohol. Thirty-six percent of men and 29% of women did not consume fruits or vegetables at least once a day. Seventy-three percent of men and 81% of women did not engage in physical activity or sports at least three times a week.

Fig 1 shows the prevalence of objectively measured HBP according to SES. As the figure shows, there is a clear SES gradient in HBP among older individuals in Colombia. Prevalence of HBP among older individuals with no formal education is 39.5%, 33% among those with primary education and 27.7% among those with secondary or post-secondary education. Furthermore, prevalence of HBP among those in the 1st assets quartile (most deprived) is 38.2%, 32.5 and 31.5% in the 2nd and 3rd quartile, and 27.3% in the 4th quartile (most affluent).

Table 2 shows the results of two multivariate logistic regression models regressing HBP on SES and covariates. Compared to the reference group with no formal education, those with primary education have an approximately 22% lower risk of suffering from HBP (OR = 0.77, P-value = 0.014). Compared to the reference group, those with secondary or post-secondary education have a 37% lower risk of suffering from HBP (OR = 0.626, P-value = <0.001). Furthermore, compared to those in the 1st quartile of assets (most deprived), being in the 2nd quartile is associated with a 24% lower risk of BHP (OR = 0.76, P-value = 0.006), while being in the 3rd quartile of assets is associated with a 28% lower risk of BHP (OR = 0.72, P-value = <0.001) and being in the 4th quartile of assets (most affluent) with a 44% lower risk of HBP (OR = 0.66, P-value = <0.001).

Results showing associations between SES and SBP and DBP (in mm Hg) are shown in S1 Appendix Supplementary Table A1.

Fig 2 shows associations between SES with additional risk factors for CVD. Detailed results are shown in S1 Appendix Supplementary Table A2.

As Fig 2 shows, in tendency, higher SES is associated with healthier behaviors in terms of smoking, fruits or vegetables consumption, and physical activity. In contrast, with regard to alcohol consumption the results show that individuals with higher SES are, on average, more likely to consume any alcohol compared to those with lower SES. No significant difference existed in terms of the likelihood of being obese according to educational achievement. However, those with a higher number of assets were significantly more likely to be obese compared to those with a lower number of assets.

## Discussion

The aim of this study was to systematically assess associations between SES and HBP as well as additional risk factors for CVD among older individuals in Colombia, drawing on nationally representative data from the SABE survey. The results show that there exists a marked SES gradient in HBP among older individuals. Thus, older individuals with higher SES have a substantially lower risk of suffering from HPB compared to those with lower SES. Having higher SES also is associated with lower likelihood of unhealthy behaviors in terms of smoking, fruits or vegetable consumption as well as physical activity. In contrast, individuals with higher SES are more likely to consume alcohol and, those with more assets, more likely to be obese.

### Relation with other studies

The results of this study—showing a consistent and marked SES gradient in HBP across all SES categories—are consistent with studies in high-income countries and a study among older individuals in Brazil [16]. However, they contrast results obtained for older individuals in Ecuador [27], adults in Peru, [19] as well as older urban residents in Chile, Uruguay, Argentina and Brazil that did not find a significant relation between SES and HBP. [28] A study of older individuals in the Brazilian state of Rio Grande do Sul only found that risk of HBP was higher among the middle SES group compared to the lowest SES group, but lower in the highest SES group compared to the lowest SES group. [29] Results for older individuals in Costa Rica have shown that no significant difference existed in terms of SBP and SES among women, while men from the highest SES group had significant lower SBP compared to the lowest SES group. [17] However, no significant difference in SBP existed between the lowest SES group and the middle SES group. Another study, also among older individuals in Costa Rica, found that in tendency individuals from the lowest SES group had lower risk of HBP, though the differences were not significant. [18]

The absence of a clear SES gradient in HBP in various LA countries may be the result of several factors. On the one hand, selection effects at higher ages due to which individuals with lower SES are less likely to survive to higher ages, may explain a lower SES gradient among older individuals. [18,30] Such selection effects may differ from country to country, as well as historical period. However, such effects cannot easily explain the relative absence of a marked SES gradient in health in Costa Rica given that live expectancy in Costa Rica, with 79.6 years, is very high in comparison. [31] On the other hand, the absence of a marked SES gradient in CVD in Ecuador as well as the Brazilian state of Rio Grande do Sul may be, at least partly, explained by systematic under-reporting of health conditions among lower SES groups. Under-reporting of HPB by lower SES groups has been documented in various studies, and may be due to the lack of access to medical care as well as poor health literacy. [32,33] Such under-reporting of HBP may systematically under-estimate the SES gradient in studies that rely on self-reports (usually based on a question asking whether the respondent had ever been diagnosed with HBP condition by a doctor), such as in the case of the study of Ecuador [27] and the Brazilian state of Rio Grande do Sul. [29]

With regard to the results for additional CVD risk factors the results of this study are in line with a systematic review on the relation between SES and risk factors for NCDs in LMICs showing that individuals with low SES are more likely to consume less fruits and vegetables, engage in less physical activity and are more likely to smoke compared to their counterparts with higher SES. [21] The findings of our study for older individuals in Colombia, however, contrast those for older individuals in Costa Rica [17] where no significant SES gradient existed for either smoking, sedentary behavior or being obese (with only some significant differences existing in terms of smoking and sedentary behaviors among women). Different to older individuals in Colombia, the absence of such associations in Costa Rica may help to explain why there also exists no SES gradient in HBP in this country. The results of our study also contrast results of a study among adults in Peru that found no SES gradient in either smoking or alcohol consumption. [19] The significant and positive association between higher SES and obesity (although only in terms of assets) among older individuals in Colombia stands in contrast to evidence from LMICs showing that obesity is more prevalent among individuals with lower SES. [34] Taken together, with the exception of obesity, the population of older individuals in Colombia appears to be already at a comparatively advanced stage of the epidemiological transition leading to a shift in the burden of NCDs from higher to lower SES groups. [35]

The existence of a substantial SES gradient in HBP and several additional risk factors for CVD among older individuals in Colombia is highly relevant in the context of the Rio Political Declaration on Social Determinants of Health. [36,37] Among other things, the declaration calls for a systematic monitoring of health inequalities. As mentioned above, evidence on health inequalities among older individuals, which bear the highest burden of many diseases, in LA is still missing for many countries in the region. Importantly, the SES gradients in CVD and its underlying risk factors among older individuals, differ greatly between LA and are by no means uniform, as highlighted by the differences in results for example between Colombia, Ecuador and Cost Rica. While HPB is a readily treatable risk factor, [38] having undetected or uncontrolled HBP is significantly more common among lower-SES groups among older Colombians. Thus, supplementary analyses (not shown) suggest that prevalence of controlled HPB among the lowest educational group in our sample is only 25%, but 35% among the highest educational group. The high prevalence of HPB among older individuals in Colombia (32%) and the marked SES gradient underscore the importance of a public health approach focused on primary prevention and management of HBP that takes into account the specific conditions of older individuals, especially among disadvantaged groups. As argued elsewhere, such an approach, in order to be successful, will have to involve different strategies for raising awareness and for improving primary prevention and access to medication. [38]

### Limitations

Some limitations should be considered. First, due to its cross-sectional nature, the results of this study only reveal associations between SES and CVD, as well as associated risk factors. In consequence, the results have no causal interpretation, i.e. they do not suggest that there would exist a causal relation between SES and health or related behaviors. Secondly, although the data from the SABE study are nationally representative, with a comparatively large sample size, information on blood pressure was only collected for a sub-sample of respondents. Respondents were randomly invited to participate in the blood pressure assessment. However, supplementary analyses reveal that the sub-sample for which data on blood pressure is available differs slightly in terms of observed characteristics from the remaining sample. With regard to average educational achievement and number of disposable household assets, the sub-sample that includes data on blood pressure is slightly more educated (with a mean level of education of 2.6 in the sub-sample, compared to 2.5 in the remainder of the sample) and has a slightly larger number of disposable assets (with a mean number of assets of 7.9 in the sub-sample, compared to 7.3 in the remainder of the sample). Although both differences are statistically significant, overall, they are relatively small. With regard to the main research question of this study, the circumstance that individuals with higher SES are slightly over-represented in the sub-sample used in this study, in tendency, should result in an under-estimation of the SES gradient in terms of health and health behaviors, rather than an over-estimation.

## Conclusions

This study is the first to assess inequality in key risk factors for CVD according to SES among a representative sample of older individuals in Colombia. In contrast to results from other LA countries, inequalities among older individuals in Colombia are substantial, revealing that individuals with lower SES bear a significantly larger burden of HBP than their counterparts with higher SES. The results emphasize that a comprehensive public health approach for CVD that aims to reduce SES inequality among older individuals should take into account specific risk factors and needs for care of disadvantaged groups.

## Supporting information

S1 Appendix (DOCX)

## References

[1] Kankeu HT, Saksena P, Xu K, Evans DB. The financial burden from non-communicable diseases in low-and middle-income countries: a literature review. Health Res Policy Syst. 2013;11(1):31. 23947294 10.1186/1478-4505-11-31 PMC3751656

[2] Perel P, Casas JP, Ortiz Z, Miranda JJ. Noncommunicable diseases and injuries in Latin America and the Caribbean: time for action. PLoS Med. 2006;3(9):e344 10.1371/journal.pmed.0030344 16953660 PMC1570186

[3] Webber L, Kilpi F, Marsh T, Rtveladze K, Brown M, McPherson K. High rates of obesity and non-communicable diseases predicted across Latin America. PloS One. 2012;7(8):e39589 10.1371/journal.pone.0039589 22912663 PMC3418261

[4] Yusuf S, Reddy S, Ôunpuu S, Anand S. Global burden of cardiovascular diseases: part I: general considerations, the epidemiologic transition, risk factors, and impact of urbanization. Circulation. 2001;104(22):2746–53. 10.1161/hc4601.099487 11723030

[5] Murray CJ, Lopez AD, World Health Organization. The global burden of disease: a comprehensive assessment of mortality and disability from diseases, injuries, and risk factors in 1990 and projected to 2020: summary. World Health Organization; 1996.

[6] Barceló A. Cardiovascular diseases in Latin America and the Caribbean. The Lancet. 2006;368(9536):625–6. 10.1016/S0140-6736(06)69223-4 16920450

[7] Fernando L, Pamela S, Alejandra L. Cardiovascular disease in Latin America: the growing epidemic. Prog Cardiovasc Dis. 2014;57(3):262–7. 10.1016/j.pcad.2014.07.007 25443823

[8] Ruilope LM, Chagas ACP, Brandão AA, Gómez-Berroterán R, Alcalá JJA, Paris JV, et al Hypertension in Latin America: Current perspectives on trends and characteristics. Hipertens Riesgo Vasc. 2017 1 1;34(1):50–6. 10.1016/j.hipert.2016.11.005 28007488

[9] Miranda JJ, Herrera VM, Chirinos JA, Gómez LF, Perel P, Pichardo R, et al Major cardiovascular risk factors in Latin America: a comparison with the United States. The Latin American consortium of studies in obesity (LASO). PloS One. 2013;8(1):e54056 10.1371/journal.pone.0054056 23349785 PMC3547948

[10] Di Cesare M, Khang Y-H, Asaria P, Blakely T, Cowan MJ, Farzadfar F, et al Inequalities in non-communicable diseases and effective responses. The Lancet. 2013;381(9866):585–97. 10.1016/S0140-6736(12)61851-0 23410608

[11] Lloyd-Sherlock P, Beard J, Minicuci N, Ebrahim S, Chatterji S. Hypertension among older adults in low-and middle-income countries: prevalence, awareness and control. Int J Epidemiol. 2014;43(1):116–28. 10.1093/ije/dyt215 24505082 PMC3937973

[12] Rosero-Bixby L, Dow WH. Exploring why Costa Rica outperforms the United States in life expectancy: A tale of two inequality gradients. Proc Natl Acad Sci. 2016;113(5):1130–7. 10.1073/pnas.1521917112 26729886 PMC4747769

[13] Mackenbach JP, Cavelaars A, Kunst AE, Groenhof F. Socioeconomic inequalities in cardiovascular disease mortality. An international study. Eur Heart J. 2000;21(14):1141–51. 10.1053/euhj.1999.1990 10924297

[14] Havranek EP, Mujahid MS, Barr DA, Blair IV, Cohen MS, Cruz-Flores S, et al Social determinants of risk and outcomes for cardiovascular disease: a scientific statement from the American Heart Association. Circulation. 2015;132(9):873–98. 10.1161/CIR.0000000000000228 26240271

[15] Alves L, Azevedo A, Silva S, Barros H. Socioeconomic inequalities in the prevalence of nine established cardiovascular risk factors in a southern European population. PloS One. 2012;7(5):e37158 10.1371/journal.pone.0037158 22666343 PMC3362583

[16] Lima-Costa MF, De Oliveira C, Macinko J, Marmot M. Socioeconomic inequalities in health in older adults in Brazil and England. Am J Public Health. 2012;102(8):1535–41. 10.2105/AJPH.2012.300765 22698020 PMC3464850

[17] Rehkopf DH, Dow WH, Rosero-Bixby L. Differences in the association of cardiovascular risk factors with education: a comparison of Costa Rica (CRELES) and the USA (NHANES). J Epidemiol Community Health. 2010;64(9):821–8. 10.1136/jech.2009.086926 19822554 PMC2976055

[18] Rosero-Bixby L, Dow WH. Surprising SES gradients in mortality, health, and biomarkers in a Latin American population of adults. J Gerontol B Psychol Sci Soc Sci. 2009;64(1):105–17. 10.1093/geronb/gbn004 19196695 PMC2654981

[19] Quispe R, Benziger CP, Bazo-Alvarez JC, Howe LD, Checkley W, Gilman RH, et al The relationship between socioeconomic status and CV risk factors: the CRONICAS cohort study of peruvian adults. Glob Heart. 2016;11(1):121–30. 10.1016/j.gheart.2015.12.005 27102029 PMC4838671

[20] Stringhini S, Bovet P. Socioeconomic status and risk factors for non-communicable diseases in low-income and lower-middle-income countries. Lancet Glob Health. 2017;5(3):e230–1. 10.1016/S2214-109X(17)30054-2 28193380

[21] Allen L, Williams J, Townsend N, Mikkelsen B, Roberts N, Foster C, et al Socioeconomic status and non-communicable disease behavioural risk factors in low-income and lower-middle-income countries: a systematic review. Lancet Glob Health. 2017;5(3):e277–89. 10.1016/S2214-109X(17)30058-X 28193397 PMC5673683

[22] Ministerio de Salud y Protección Social de Colombia. SABE Colombia 2015: Estudio Nacional de Salud, Bienestar y Envejecimiento [Internet]. 2015. https://www.minsalud.gov.co/sites/rid/Lists/BibliotecaDigital/RIDE/VS/ED/GCFI/Resumen-Ejecutivo-Encuesta-SABE.pdf

[23] Lenis DO, Paz FM. Survey on Health, Well-being and Aging. SABE Colombia 2015: Technical report. Colomb Médica. 2019;50(2):128–38. 10.25100/cm.v50i2.4557 PMC6774577 31607769

[24] Ocampo-Chaparro JM, Reyes-Ortiz CA, Castro-Flórez X, Montes JFG. Frailty in older adults and their association with social determinants of Health. The SABE Colombia Study. Colomb Médica. 2019;50(2):89–101. 10.25100/cm.v50i2.4121 PMC6774581 31607766

[25] Gomez F, Corchuelo J, Curcio C-L, Calzada M-T, Mendez F. SABE Colombia: Survey on Health, Well-Being, and Aging in Colombia—study design and protocol. Curr Gerontol Geriatr Res. 2016;2016. 10.1155/2016/7910205 PMC5124445 27956896

[26] Centro Nacional de Investigación en Evidencia y Tecnologías en Salud. Guía de práctica clínica: Hipertensión arterial primaria (hta) [Internet]. Bogotá: Ministerio de Salud y Protección Socia de la República de Colombia; 2013. https://www.minsalud.gov.co/sites/rid/Lists/BibliotecaDigital/RIDE/INEC/IETS/GPC_Completa_HTA.pdf

[27] Guevara PE, Andrade FCD. Socioeconomic and lifestyle factors associated with chronic conditions among older adults in Ecuador. Rev Panam Salud Pública. 2015;38:226–32. 26758001

[28] Jones A, Mitchel D, Goza F. Lifecourse socioeconomic status and cardiovascular illness in Latin America. Curr Sociol. 2014 7 8;62(7):1055–78.

[29] Fillenbaum GG, Blay SL, Pieper CF, King KE, Andreoli SB, Gastal FL. The association of health and income in the elderly: Experience from a southern state of Brazil. PloS One. 2013;8(9):e73930 10.1371/journal.pone.0073930 24058505 PMC3772829

[30] Crimmins EM. Socioeconomic differentials in mortality and health at the older ages. Genus. 2005;163–76.

[31] Rosero-Bixby L, Dow WH. Surprising SES Gradients in Mortality, Health, and Biomarkers in a Latin American Population of Adults. J Gerontol Ser B. 2009;64B(1):105–17. 10.1093/geronb/gbn004 PMC2654981 19196695

[32] Lloyd-Sherlock P. Old age and poverty in developing countries: new policy challenges. World Dev. 2000;28(12):2157–68.

[33] Lloyd-Sherlock P. Social policy and population ageing: challenges for north and south. Int J Epidemiol. 2002;31(4):754–7. 10.1093/ije/31.4.754 12177014

[34] Dinsa GD, Goryakin Y, Fumagalli E, Suhrcke M. Obesity and socioeconomic status in developing countries: a systematic review. Obes Rev. 2012;13(11):1067–79. 10.1111/j.1467-789X.2012.01017.x 22764734 PMC3798095

[35] Stringhini S, Bovet P. Commentary: The social transition of cardiovascular disease in low-and middle-income countries: wait and see is not an option. Int J Epidemiol. 2013;42(5):1429–31. 10.1093/ije/dyt084 24008332

[36] Lee JH, Sadana R, Health C on SD of. Improving equity in health by addressing social determinants. Geneva: World Health Organization; 2011.

[37] Organization WH. Rio political declaration on social determinants of health. In 2011. p. 19–21.

[38] Lloyd-Sherlock P, Beard J, Minicuci N, Ebrahim S, Chatterji S. Hypertension among older adults in low-and middle-income countries: prevalence, awareness and control. Int J Epidemiol. 2014;43(1):116–28. 10.1093/ije/dyt215 24505082 PMC3937973

