# Supplementary material for: Socio-economic inequalities in high blood pressure and additional risk factors for cardiovascular disease among older individuals in Colombia: Results from a nationally representative study
Source: PLoS One. 2020 Jun 9;15(6):e0234326. doi: 10.1371/journal.pone.0234326 (PMC7282633; doi:10.1371/journal.pone.0234326)
Supplement: S1 Appendix — (DOCX) [file pone.0234326.s001.docx]

Supplementary Appendix

**Socio-economic inequalities in high blood pressure and additional risk factors for cardiovascular disease among older individuals in Colombia: results from a nationally representative study**

| **Supplementary Table A1. Ordinary Least Square Regressions of Blood Pressure on Socio-Economic Status among older Individuals in Colombia** | | | | | | | |
| --- | --- | --- | --- | --- | --- | --- | --- |
|  |  |  |  |  |  |  |  |
|  | **Systolic blood pressure (SBP) in mm Hg** | | | | | | |
|  |  |  |  |  |  |  |  |
|  | **Education** | | |  | **Assets** | | |
|  |  |  |  |  |  |  |  |
|  | *Coefficient* | *95% CI* | *P-value* |  | *Coefficient* | *95% CI* | *P-value* |
|  |  |  |  |  |  |  |  |
| **Education** |  |  |  |  |  |  |  |
| None (reference) |  |  |  |  |  |  |  |
| Primary | -2.449 | (-4.856, -0.042) | 0.046 |  |  |  |  |
| Secondary or Post-Secondary | -5.391 | (-8.010, -2.773) | <0.001 |  |  |  |  |
|  |  |  |  |  |  |  |  |
| **Assets** |  |  |  |  |  |  |  |
| Quartile 1 (most deprived) (reference) |  |  |  |  |  |  |  |
| Quartile 2 |  |  |  |  | -3.338 | (-5.564, -1.112) | 0.003 |
| Quartile 3 |  |  |  |  | -4.739 | (-6.812, -2.666) | <0.001 |
| Quartile 4 (most affluent) |  |  |  |  | -6.849 | (-9.031, -4.666) | <0.001 |
|  |  |  |  |  |  |  |  |
| Joint significance test for SES | <0.0001 | | |  | <0.0001 | | |

|  |  |  |  |  |  |  |  |
| --- | --- | --- | --- | --- | --- | --- | --- |
|  | **Diastolic blood pressure (DBP) in mm Hg** | | | | | | |
|  |  |  |  |  |  |  |  |
|  | **Education** | | |  | **Assets** | | |
|  |  |  |  |  |  |  |  |
|  | *Coefficient* | *95% CI* | *P-value* |  | *Coefficient* | *95% CI* | *P-value* |
|  |  |  |  |  |  |  |  |
| **Education** |  |  |  |  |  |  |  |
| None (reference) |  |  |  |  |  |  |  |
| Primary | -2.449 | (-4.856, -0.042) | 0.046 |  |  |  |  |
| Secondary or Post-Secondary | -5.391 | (-8.010, -2.773) | <0.001 |  |  |  |  |
|  |  |  |  |  |  |  |  |
| **Assets** |  |  |  |  |  |  |  |
| Quartile 1 (most deprived) (reference) |  |  |  |  |  |  |  |
| Quartile 2 |  |  |  |  | -3.338 | (-5.564, -1.112) | 0.003 |
| Quartile 3 |  |  |  |  | -4.739 | (-6.812, -2.666) | <0.001 |
| Quartile 4 (most affluent) |  |  |  |  | -6.849 | (-9.031, -4.666) | <0.001 |
|  |  |  |  |  |  |  |  |
| Joint significance test for SES | <0.0001 | | |  | <0.0001 | | |
| Abbreviations: OR=odds ratio; CI=confidence interval; SES=socio-economic status; mm Hg=millimeters of mercury. | | | | | | | |
| Notes: SBP and DBP was operationalized as the average of the second and third measurements of the respondent’s right arm. | | | | | | | |

| **Supplementary Table A2. Logistic Regressions of Obesity and Risk Factors for Cardiovascular Disease on Socio-Economic Status among older Individuals in Colombia** | | | | | | | | |
| --- | --- | --- | --- | --- | --- | --- | --- | --- |
|  |  |  |  |  |  |  |  |  |
| **Obese (yes)** | | | | | | | | |
|  |  |  |  |  |  |  |  |  |
|  | *OR* | *95% CI* | *P-value* |  |  | *OR* | *95% CI* | *P-value* |
|  |  |  |  |  |  |  |  |  |
| **Education** |  |  |  |  | **Assets** |  |  |  |
| None (reference) |  |  |  |  | Quartile 1 (most deprived) (reference) | | |  |
| Primary | 0.98 | (0.771, 1.245) | 0.867 |  | Quartile 2 | 1.307 | (1.030, 1.657) | 0.027 |
| Secondary or Post-Secondary | 0.911 | (0.697, 1.190) | 0.494 |  | Quartile 3 | 1.477 | (1.185, 1.840) | 0.001 |
|  |  |  |  |  | Quartile 4 (most affluent) | 1.696 | (1.351, 2.129) | <0.001 |
| **Smoker (yes)** | | | | | | | | |
|  |  |  |  |  |  |  |  |  |
|  | *OR* | *95% CI* | *P-value* |  |  | *OR* | *95% CI* | *P-value* |
|  |  |  |  |  |  |  |  |  |
| **Education** |  |  |  |  | **Assets** |  |  |  |
| None (reference) |  |  |  |  | Quartile 1 (most deprived) (reference) | | |  |
| Primary | 0.687 | (0.509, 0.926) | 0.014 |  | Quartile 2 | 0.760 | (0.565, 1.023) | 0.070 |
| Secondary or Post-Secondary | 0.728 | (0.522, 1.016) | 0.062 |  | Quartile 3 | 0.735 | (0.558, 0.967) | 0.028 |
|  |  |  |  |  | Quartile 4 (most affluent) | 0.689 | (0.514, 0.924) | 0.013 |
|  |  |  |  |  |  |  |  |  |
| **Alcohol consumption (yes)** | | | | | | | | |
|  |  |  |  |  |  |  |  |  |
|  | *OR* | *95% CI* | *P-value* |  |  | *OR* | *95% CI* | *P-value* |
|  |  |  |  |  |  |  |  |  |
| **Education** |  |  |  |  | **Assets** |  |  |  |
| None (reference) |  |  |  |  | Quartile 1 (most deprived) (reference) | | |  |
| Primary | 1.03 | (0.749, 1.415) | 0.856 |  | Quartile 2 | 0.944 | (0.695, 1.282) | 0.713 |
| Secondary or Post-Secondary | 1.60 | (1.141, 2.238) | 0.006 |  | Quartile 3 | 1.412 | (1.079, 1.848) | 0.012 |
|  |  |  |  |  | Quartile 4 (most affluent) | 1.673 | (1.271, 2.204) | <0.001 |
|  |  |  |  |  |  |  |  |  |

| **Fruits or vegetables consumption (no)** | | | | | | | | |
| --- | --- | --- | --- | --- | --- | --- | --- | --- |
|  |  |  |  |  |  |  |  |  |
|  | *OR* | *95% CI* | *P-value* |  |  | *OR* | *95% CI* | *P-value* |
|  |  |  |  |  |  |  |  |  |
| **Education** |  |  |  |  | Quartile 1 (most deprived) (reference) | | |  |
| None (reference) |  |  |  |  | Quartile 2 | 0.774 | (0.639, 0.936) | 0.008 |
| Primary | 0.404 | (0.320,0.510) | <0.001 |  | Quartile 3 | 0.499 | (0.415, 0.601) | <0.001 |
| Secondary or Post-Secondary | 0.701 | (0.612, 0.804) | <0.001 |  | Quartile 4 (most affluent) | 0.369 | (0.301, 0.451) | <0.001 |
|  |  |  |  |  |  |  |  |  |
| **Physical activity (no)** | | | | | | | | |
|  |  |  |  |  |  |  |  |  |
|  | *OR* | *95% CI* | *P-value* |  |  | *OR* | *95% CI* | *P-value* |
|  |  |  |  |  |  |  |  |  |
| **Education** |  |  |  |  | **Assets** |  |  |  |
| None (reference) |  |  |  |  | Quartile 1 (most deprived) (reference) | | |  |
| Primary | 0.655 | (0.492, 0.872) | 0.004 |  | Quartile 2 | 0.862 | (0.672, 1.105) | 0.241 |
| Secondary or Post-Secondary | 0.311 | (0.230, 0.419) | <0.001 |  | Quartile 3 | 0.592 | (0.474, 0.739) | <0.001 |
|  |  |  |  |  | Quartile 4 (most affluent) | 0.412 | (0.329, 0.517) | <0.001 |
| Abbreviations: OR=odds ratio; CI=confidence interval; SES=socio-economic status. | | | | | | | | |
| Notes: Authors' own calculations based on data from the Encuesta de Salud, Bienestar y Envejecimiento (SABE) study including individuals aged 60 years or above. Results are shown in terms of OR and refer to the difference in relative risk of each category in comparison with the lowest category in terms of either education or assets. Results were obtained from multivariate logistic regressions that controlled for age, gender and region of residence (not shown). | | | | | | | | |
